# Recent Advances in Simulation Studies on the Protein Corona

**DOI:** 10.3390/pharmaceutics16111419

**Published:** 2024-11-06

**Authors:** Hwankyu Lee

**Affiliations:** Department of Chemical Engineering, Dankook University, Yongin-si 16890, Republic of Korea; leeh@dankook.ac.kr

**Keywords:** protein corona, molecular dynamics simulation, protein–nanoparticle interaction, protein–protein interaction, drug delivery

## Abstract

When flowing through the blood stream, drug carriers such as nanoparticles encounter hundreds of plasma proteins, forming a protein layer on the nanoparticle surface, known as the “protein corona”. Since the protein corona influences the size, shape, and surface properties of nanoparticles, it can modulate their circulating lifetime, cytotoxicity, and targeting efficiency. Therefore, understanding the mechanism of protein corona formation at the atomic scale is crucial, which has become possible due to advances in computer power and simulation methodologies. This review covers the following topics: (1) the structure, dynamics, and composition of protein corona on nanoparticles; (2) the effects of protein concentration and ionic strength on protein corona formation; (3) the effects of particle size, morphology, and surface properties on corona formation; (4) the interactions among lipids, membranes, and nanoparticles with the protein corona. For each topic, mesoscale, coarse-grained, and all-atom molecular dynamics simulations since 2020 are discussed. These simulations not only successfully reproduce experimental observations but also provide physical insights into the protein corona formation. In particular, these simulation findings can be applied to manipulate the formation of a protein corona that can target specific cells, aiding in the rational design of nanomedicines for drug delivery applications.

## 1. Introduction

Natural or synthetic nanoparticles such as liposomes, hydrogels, dendrimers, nanosheets, and polymeric and metallic nanoparticles have been widely studied for delivering genes, drugs, or sensing molecules to specific cells [1,2,3,4,5,6,7,8,9]. When these nanoparticles pass through the bloodstream, they encounter hundreds of plasma proteins, leading to the formation of a protein layer on the nanoparticle surface called the “protein corona” [10,11,12]. The protein corona consists of a hard corona (irreversible binding between proteins and the nanoparticle surface, forming the first layer adjacent to the nanoparticle) and a soft corona (reversible binding between proteins, forming a low-density protein layer between the hard corona and the water region) [10,11]. Protein corona modulates the size and surface properties of nanoparticles, thereby influencing their circulating lifetime, cytotoxicity, cellular uptake, and targeting efficiency, which can either enhance or reduce drug delivery efficiency [13,14,15,16]. For instance, the protein corona can guide nanoparticles toward specific cell surfaces composed of receptors with a high affinity for the protein corona [17]. To achieve this, nanoparticles can be pre-coated with a specific protein in vitro, which directs subsequent protein adsorption in vivo through protein–protein interactions, resulting in a dual-layered protein corona structure that influences the fate of nanoparticles in the body [18].

**Figure 1:**
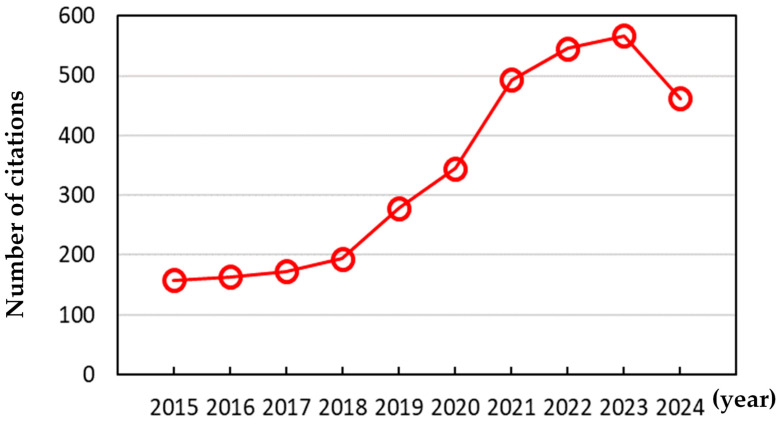
The number of citations of research articles on the topic of the protein corona studied using molecular dynamics simulations as of September/2024.

To control protein corona formation, nanoparticles are often modified by grafting hydrophilic polymers such as polyethylene glycol (PEG), which sterically shield nanoparticles from plasma proteins [19,20]. However, grafting PEG at high concentrations destabilizes nanoparticle stability and blocks ligand molecules, limiting the amount of PEG that can be grafted [21,22,23]. Therefore, PEGylation cannot entirely prevent protein corona formation, making it essential to understand this process. Although experiments have characterized the structure and composition of the protein corona, interpreting atomic-level phenomena and fundamental interactions is not always easy due to the limited resolution of experimental methodologies, which has motivated many theoretical and simulation studies. In particular, molecular dynamics (MD) simulations have been applied to capture the conformation and dynamics of plasma proteins adsorbed onto nanoparticles, uncovering their competitive adsorption and desorption [24,25,26,27,28,29,30,31,32,33,34], as described by the Vroman effect [35]. Figure 1 shows that citations for simulation studies regarding the protein corona have drastically increased over the past five years. Note that for 2024, citation numbers are recorded only up to September. 

In this review, we focus on MD simulation research on nanoparticles with a protein corona, particularly over the past five years. For simulation work prior to 2020, previous review papers are highly recommended [36,37,38]. Here, we review the structure, dynamics, and composition of the protein corona on nanoparticles (Section 2), the effects of protein concentration and ionic strength on protein corona formation (Section 3), the effects of particle size, morphology, and surface properties on protein corona formation (Section 4), and interactions among lipids, membranes, and nanoparticles with a protein corona (Section 5). In each section, all-atom, coarse-grained (CG), and mesoscale simulations are reviewed separately to highlight their specific roles at different simulation scales.

## 2. Structure, Dynamics, and Composition of Protein Corona on Nanoparticles

The structure, dynamics, and composition of plasma proteins change upon adsorption onto nanoparticles, driven by competitive nanoparticle–protein and protein–protein interactions, leading to larger contact areas [39]. To understand this, major plasma proteins have been simulated with various nanoparticles such as gold, polystyrene, graphene oxide, zinc oxide, molybdenum disulfide, carbon nanotubes, zeolite, and iron surfaces. Since capturing structural changes in proteins is crucial, most simulations have employed all-atom models, although some CG simulations have also provided important physical insights at larger scales.

### 2.1. Mesoscopic and Coarse-Grained Simulations

Tavanti and Menziani simulated the adsorption of serum albumin (SA), hemoglobin, antiproteinase, and complement C3 onto gold nanoparticles, showing that gold nanoparticles bound more strongly to complement C3 than to the other proteins. Proteins bound to gold nanoparticles via strong hydrophobic interactions, adapting to the nanoparticle surface without significant changes to their secondary structures, in agreement with experimental findings showing minimal conformational changes in SA on gold nanoparticles with a 4.5 nm diameter [40]. Tavakol et al. explored the adsorption of immunoglobulin (IgG) and SA proteins onto graphene oxide surfaces, showing that SA adsorption was more likely to occur after IgG was adsorbed rather than the reverse. The higher lateral diffusion of SA allowed for a more stable configuration, while initial SA adsorption restricted the lateral diffusion of IgG, suggesting that SA could replace IgG on the graphene oxide surface [41].

### 2.2. All-Atom Simulations

**Figure 2:**
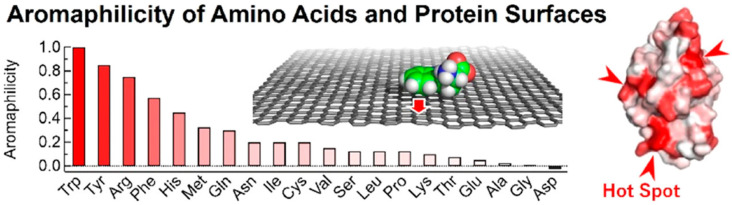
Aromaphilicity index of amino acids and protein surfaces in relation to protein binding affinity for carbon nanomaterials. Reproduced with permission from [44], American Chemical Society, 2021.

Our group explored the adsorption of plasma proteins onto 10 nm sized polystyrene (PS) particles with varying charges (cationic, anionic, and neutral) by calculating binding free energies, showing that proteins bound to PS particles irrespective of their charge, consistent with experimental observations showing the adsorption of anionic proteins onto both cationic and anionic particles. In particular, simulations of mixed protein systems revealed competitive adsorption, forming protein layers that affected protein mobility based on their position in the layer, which provided insight into the early stages of protein corona formation and differences in binding strength between inner and outer layers and supported the Vroman effect [42]. Hassanian et al. observed the structural change in SA adsorbed onto zinc oxide (ZnO) nanoparticles mainly via electrostatic interactions between charged amino acids of SA and ZnO nanoparticles, leading to the reduced compactness of the SA protein [43]. Hirano and Kameda calculated binding free energies between 20 individual amino acids and carbon nanomaterial surfaces such as carbon nanotube (CNT) or graphene, showing that arginine and aromatic amino acids preferably bound to those carbon nanomaterials (Figure 2), to an extent dependent on the surface curvature but not on the chiral angle, consistent with the experimental study using a CNT-immobilized liquid chromatographic column [44].

**Figure 3:**
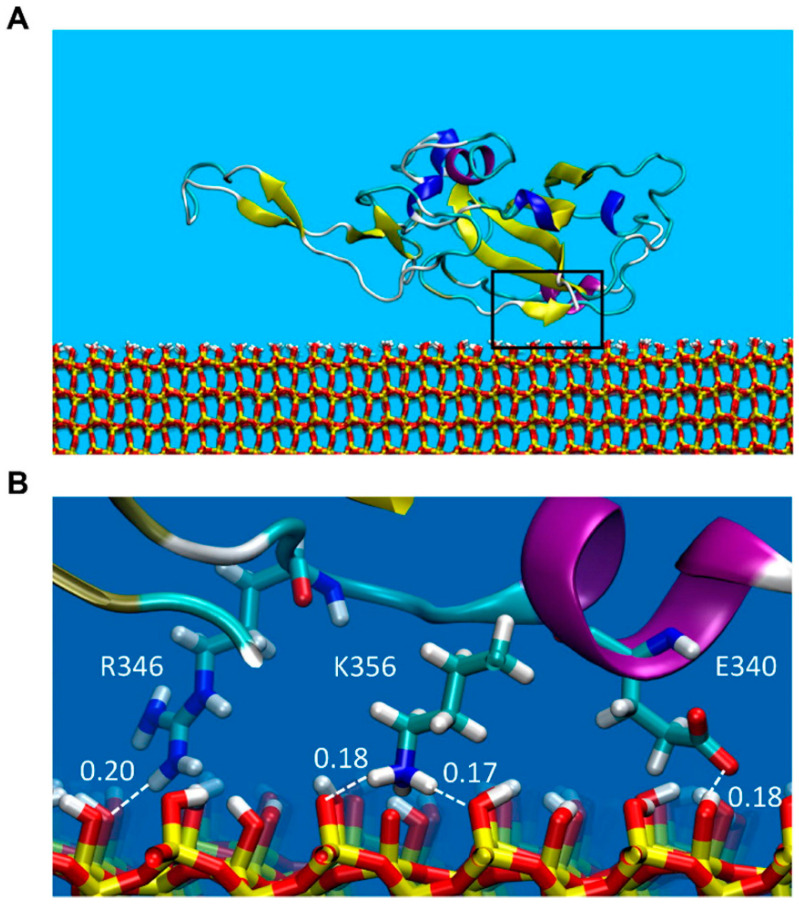
(**A**) Adsorption of SARS-CoV-2 receptor-binding domain (RBD) onto the silica surface. (**B**) Formation of hydrogen bonds between three residues (R346, K356, and E340) of SARS-CoV-2 RBD and the silica surface. Reproduced with permission from [49], Elsevier Ltd., 2022.

Cao et al. observed stronger hydrogen bonding and salt bridge interactions of molybdenum disulfide (MoS_2_) nanodots with fibrinogen (FG) than with SA and apolipoprotein E [45]. Jomhori et al. observed that single-walled carbon nanotubes (SWCNTs) improved the stability of SARS-CoV-2’s spike glycoprotein by reducing internal hydrogen bonds and enhancing solvent-accessible surface, without altering the protein’s secondary structure [46]. Wang et al. simulated SA, IgG, and FG adsorbed onto gold nanoparticles and observed the structural rearrangements of proteins because of reduced internal energy and increased hydrogen bonds, with a shift from α-helical to β-sheet content to balance the high interfacial energy [47]. Yin et al. found that the binding strengths between plasma proteins and the SARS-CoV-2 receptor-binding domain (RBD) were in the order of SA > apolipoprotein E > IgG. In particular, the adsorption of apolipoprotein E led to a significant structural change in SARS-CoV-2 RBD, potentially allowing the virus to hijack its metabolic pathway for cell entry [48]. Soloviev et al. observed that the primary interaction between the SARS-CoV-2 RBD and silica nanoparticle surface was driven by electrostatic interactions between the cationic patch and partially ionized hydroxyl groups, which was further supported by hydrogen bonds (Figure 3) [49].

**Figure 4:**
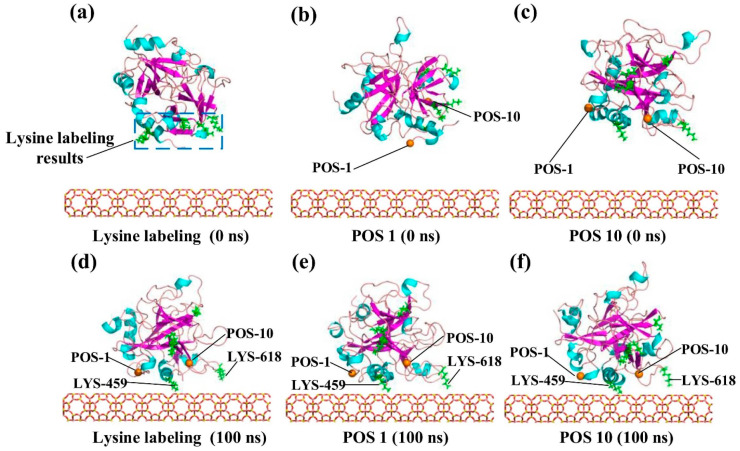
Conformations of thrombin bound to CaA zeolite: (**a**) lysine labeling, (**b**,**c**) different initial positions, and (**d**–**f**) final configurations. Reproduced with permission from [52], Elsevier Ltd., 2023.

Al-Badri et al. found that graphene-oxide (GO) functionalization denatured apolipoprotein-cIII (apo-c3) due to strong electrostatic interactions, leading to protein aggregation. GO adsorption exposed hydrophobic residues, stabilizing the protein backbone with β-bridges, while double-functionalized GO (C2GO) preserved apo-c3’s structure through weaker van der Waals (vdW) interactions [50]. Wu et al. simulated monomers, dimers, and oligomers of human islet amyloid peptide (hIAPP) adsorbed onto MoS_2_ nanosheets, showing the strong binding between peptides and MoS_2_ nanosheets via vdW and electrostatic forces, which was verified by experiments showing the fibrillation of hIAPP on MoS_2_ nanosheets. This binding was stabilized by forming β-sheet structure, which promoted further protein–protein interactions on the MoS_2_ nanosheet [51]. Li et al. simulated the adsorption of thrombin to Ca^2+^-exchanged Linda type A (CaA-LTA) zeolite, showing that thrombin quickly adjusted its orientation and adsorbed onto the zeolite surface, with small fluctuations in adsorption distance and dipole angle afterward (Figure 4). Cationic lysine and arginine residues strongly interacted with anionic CaA surface, indicating the role of electrostatic interactions in thrombin adsorption, in agreement with an experimental dimethyl labeling analysis [52].

**Figure 5:**
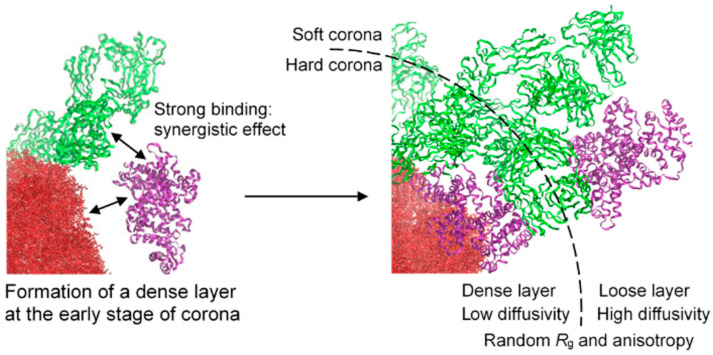
Differences in protein density and diffusivity between hard and soft coronas. The already adsorbed protein on the nanoparticle surface facilitates the additional adsorption of other proteins. Reproduced with permission from [53], Royal Society of Chemistry, 2023.

Our group simulated 6~30 SA and IgG proteins adsorbed to the 10 nm sized polystyrene nanoparticles, showing that over half of proteins densely concentrated within 3 nm of the particle surface, forming a “hard corona”, while others were more loosely distributed between 3 and 9 nm, forming a “soft corona”, in agreement with experimental observations regarding the formation of a dense protein layer adjacent to the particle surface (hard corona) and a loose protein network (soft corona). Protein conformation and diffusivity depended on their position and the electrostatics of proteins and particles. In particular, free-energy calculations showed that while charge did not significantly alter protein–particle binding strength, it influenced protein distribution, with already adsorbed proteins enhancing further binding and interactions (Figure 5) [53].

Mosaddeghi et al. performed both all-atom and coarse-grained simulations to analyze the corona formation of cow milk proteins on iron surfaces and rank these proteins by calculating binding energies, showing the prediction of protein corona composition on curved and flat iron surfaces using a competitive adsorption model [54]. Meesaragandla examined the adsorption of SA and transferrin onto a polystyrene surface, showing that both proteins rapidly adsorbed and became immobilized on the surface, primarily through hydrophobic interactions involving side chains of amino acids such as alanine, histidine, threonine, and valine. The adsorption strength was influenced by the protein’s surface composition and the clustering of hydrophobic amino acids, with transferrin showing stronger interactions through proline and leucine, which are less common in SA [55].

## 3. Effects of Protein Concentration and Ionic Strength on Protein Corona Formation

Experiments have shown the effects of protein concentration and ionic strength on nanoparticle aggregation and protein corona formation [56,57,58], although the underlying mechanism remains unclear. Understanding this mechanism requires accounting for protein–protein and particle–protein interactions at near-atomic scale. Different molar ratios of proteins and nanoparticles, which require relatively large-scale simulations, have been mostly studied using CG models. Meanwhile, ionic effects, which necessitate accurate modeling of charge interactions, have been primarily simulated using all-atom models.

### 3.1. Mesoscopic and Coarse-Grained Simulations

**Figure 6:**
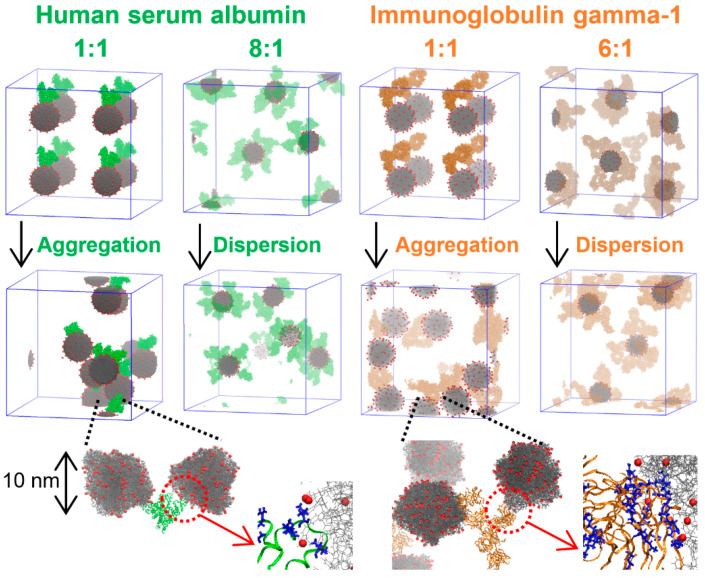
Hydrodynamics and aggregation of protein–nanoparticle complexes depend on protein type and concentration, as simulated using all-atom and coarse-grained (CG) models. Reproduced with permission from [60], WILEY-VCH, 2024.

Qin et al. simulated the adsorption of lysozyme onto silica nanoparticles, showing that increasing lysozyme concentration enhanced the conformational stability of adsorbed lysozyme, with ringlike and dumbbell-like aggregations reducing conformational loss. Higher ionic strength reduced lysozyme conformation changes and accelerated aggregation [59]. Our group simulated 10 nm sized anionic nanoparticles complexed with SA and IgG proteins at various ratios, showing that significantly larger hydrodynamic radii for individual particles at low protein concentrations (a protein-to-particle ratio of one) compared to higher concentrations or in the absence of proteins, favorably compared with experimental results showing the formation of large aggregates of anionic particles at low or intermediate protein concentrations, but not at high protein concentrations. This indicated that particle aggregation only occurred at low protein concentrations, which were driven by both electrostatic and hydrophobic interactions between particles and proteins, with variations depending on the specific proteins involved (Figure 6). In addition, we also performed all-atom simulations, showing that IgG proteins induced particle aggregation both with and without salt, whereas SA proteins only promoted aggregation in the presence of salt that weakened the electrostatic repulsion between anionic particles [60].

### 3.2. All-Atom Simulations

**Figure 7:**
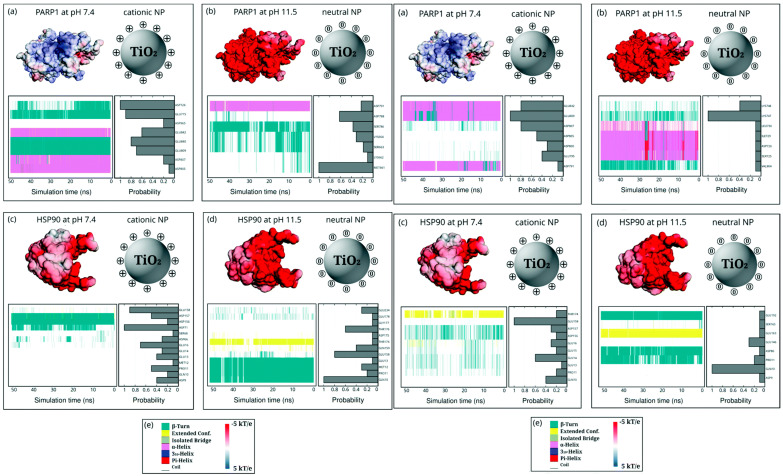
Secondary structure and probability density of amino acids in PARP1 and HSP90 proteins adsorbed onto neutral and cationic nanoparticles at pH 7.4 and 11.5, without salt (left (**a**–**e**)) or with salt (0.15 M KCl; right (**a**–**e**)). (**a**) PARP1 and cationic nanoparticle complex at pH 7.4, (**b**) PARP1 and neutral nanoparticle complex at pH 11.5, (**c**) HSP90 and cationic nanoparticle complex at pH 7.4, and (**d**) HSP90 and neutral nanoparticle complex at pH 11.5. (**e**) Colors for secondary structures and energies of proteins. Reproduced with permission from [63], Royal Society of Chemistry, 2022.

Sanchez-Guzman et al. simulated the adsorption of oxyhemoglobin onto a silica surface under ionic conditions with a pH of 7 and 9 at different temperatures of 295~400 K, showing that silica nanoparticles stabilized partially unfolded proteins through enthalpy-driven molecular interactions [61]. Li et al. also observed that MoS_2_ quantum dots bound to amyloid-beta monomers through hydrophilic interactions with the peptide’s N-terminus, while amyloid-beta oligomers and fibrils were coated by quantum dots in a “testudo-like” reverse protein corona formation, consistent with the experimental observation showing stronger hydrophilic interactions than hydrophobic interactions [62]. Siani and Di Valentin simulated the binding between dopamine-functionalized TiO_2_ nanoparticles and proteins overexpressed in cancer cells such as poly (ADP-ribose) polymerase 1 (PARP1) and heat shock protein (HSP90), showing that a less acidic intracellular pH under cytosolic ionic conditions strengthened the interactions between PARP1 and nanoparticles but weakened those between HSP90 and nanoparticles, which was interpreted by electrostatic and vdW contributions (Figure 7) [63].

**Figure 8:**
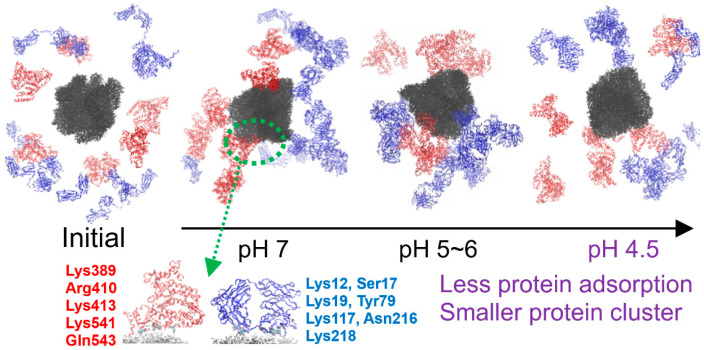
Adsorption and cluster formation of proteins at various protonation states of nanoparticles and proteins, mimicking extracellular and intracellular pH conditions. Starting from the initial random configuration of nine proteins (a mixture of 6 SA (red) and 3 IgG (blue)) around the polystyrene nanoparticle (black) (left snapshot), the final self-assembled configurations are shown for pH 7 (2nd snapshot), pH 5~6 (3rd snapshot), and pH 4.5 (4th snapshot), showing that fewer proteins adsorb to the particle at lower pH. Amino acids of the binding sites of SA and IgG at pH 7 are highlighted. Reproduced with permission from [64], Royal Society of Chemistry, 2024.

Our group performed free-energy calculations for the adsorption of SA and IgG onto 10 nm sized carboxyl-terminated polystyrene nanoparticles at various pH levels, showing that at pH 4.5, the binding between particles and proteins was significantly weakened, in agreement with experiments showing protein corona separation from nanoparticles at this acidic pH. Simulations with mixtures of multiple proteins and a particle also showed reduced protein adsorption and cluster formation at pH 4.5, which was caused by weakened electrostatic interactions due to protonation of both particles and proteins (Figure 8) [64].

## 4. Effects of Particle Size, Morphology, and Surface Properties on Corona Formation

Particle size, shape, curvature, and surface properties are known to influence protein corona formation [65,66,67], which has motivated many simulation studies, showing that protein corona formation is influenced not by any single factor but by a combination of hydrophobic, electrostatic, and vdW forces. The particle size (i.e., surface curvature) influences the adsorption and binding strength of proteins to the particle, as well as the structural changes in adsorbed proteins, with these effects depending on the specific electrostatic properties of nanoparticles and proteins involved, as reviewed in this section. In particular, nanoparticle surfaces have recently been modified by attaching charged or hydrophilic molecules that can attract specific plasma proteins, thereby controlling the composition of the protein corona [68,69,70,71].

### 4.1. Mesoscopic and Coarse-Grained Simulations

**Figure 9:**
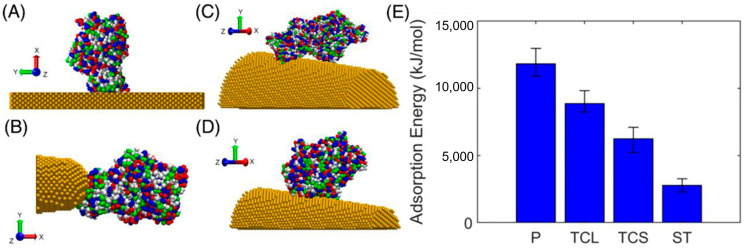
Adsorption configurations with the maximum adsorption energy for SA adsorbed onto gold nanoparticles with different curvatures: (**A**) plane (P) model; (**B**) spherical tip (ST) model; (**C**) truncated cone large (TCL) model; (**D**) truncated cone small (TCS) model; (**E**) average adsorption energy for each model. Reproduced with permission from [76], WILEY-VCH, 2023.

Jahan Sajib et al. investigated the protein corona formation of ovispirin-1 and lysozyme on gold nanoparticles, interpreted by protein–surface and protein–protein interactions, and surface hydrophobic effects. Corona structures depend on protein type and nanoparticle size, with ovispirin forming a homogeneous single layer, while lysozyme forms inhomogeneous multilayer aggregates. Smaller nanoparticles increased the angular freedom for protein adsorption orientation [72]. Rouse and Lobaskin developed a rate-equation model to describe the formation of a protein corona around nanoparticles, showing that the geometry (spherical or cylindrical shape) and size of the nanoparticle influenced the composition of the protein corona, which occurred independently of the rate constants for protein adsorption and desorption [73]. Sarker et al. simulated ovispirin-1 peptides adsorbed onto silver nanoparticles of different sizes (3.2 and 10 nm) using both all-atom and coarse-grained models, showing that peptides bound to the particle surface via hydrophilic interactions, and the secondary structures and orientations of adsorbed peptides changed, leading to larger contact areas between peptides and the particle surface. Smaller silver nanoparticles resulted in faster adsorption, denser peptide packing, and more varied orientations, with moderate changes in the peptide secondary structure and surface amino acid distribution [74]. Chen et al. investigated the dependence of ovispirin-1 peptide adsorption on the charge distribution of gold nanoparticles, showing that grafting zwitterionic peptide chains onto a gold nanoparticle reduced electrostatic and hydrophobic interactions, which slowed protein corona formation by 27% compared to bare nanoparticles. Ovispirin-1 peptides were more tilted on the zwitterionic surface compared to the bare nanoparticle [75]. Tukova et al. explored the dependence of SA adsorption on gold nanoparticle surface curvatures such as plane, large, and small truncated cones, and a spherical tip (Figure 9). In adsorption energy calculations, as the curvature of the gold surface increased, the number of interacting gold atoms and vdW forces decreased, resulting in a lower adsorption energy, which implied that SA was less likely to adsorb on highly curved surfaces [76]. Zhu et al. simulated 16 plasma proteins interacting with three different lipoplexes having different surface charges, showing that coating liposomes with hyaluronic acid and its octanoylated derivatives significantly altered the protein corona’s affinity and composition, primarily by changing the surface charge of liposomes [77].

### 4.2. All-Atom Simulations

**Figure 10:**
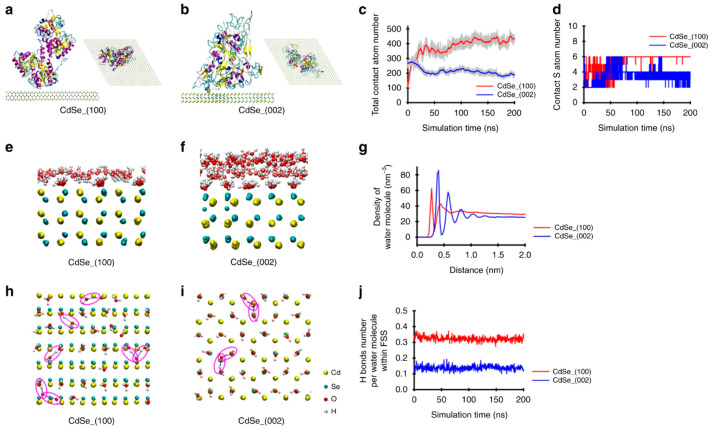
(**a**,**b**) Conformations of transferrin bound to the (100) and (002) facets of cadmium selenide (CdSe) surfaces, obtained from MD simulations. (**c**) Total number of contact atoms. (**d**) Number of contact sulfur atoms. (**e**,**f**) Formation of water molecule layers. (**g**) Density of water molecules along the z-direction. (**h**,**i**) Hydrogen bond formation in the first solvation shell of CdSe surfaces. Hydrogen bonds are highlighted in purple circles. (**j**) Number of hydrogen bonds between water and CdSe surfaces. Reproduced with permission from [79], Springer Nature, 2020.

Taha and Lee performed Monte Carlo and MD simulations to examine interactions of alanine peptide residues with various gold nanoparticles in different shapes, and utilized density-functional theory and natural bond orbital analysis to compare binding energies in gas and water phases, showing the alanine side-chain methyl group enhancing peptide interaction with gold nanoparticles [78]. Qi et al. compared adsorption energies of transferrin on (100) and (002) facets of cadmium selenide (CdSe) surfaces, showing the stronger interactions of the interfacial water with the (002) facet than with the (100) facet and thus allowing transferrin to more strongly bind to the (100) facet (Figure 10), which implied that this facet-dependent binding was influenced by different affinities of crystal facets to water molecules in the first solvation shell [79].

**Figure 11:**
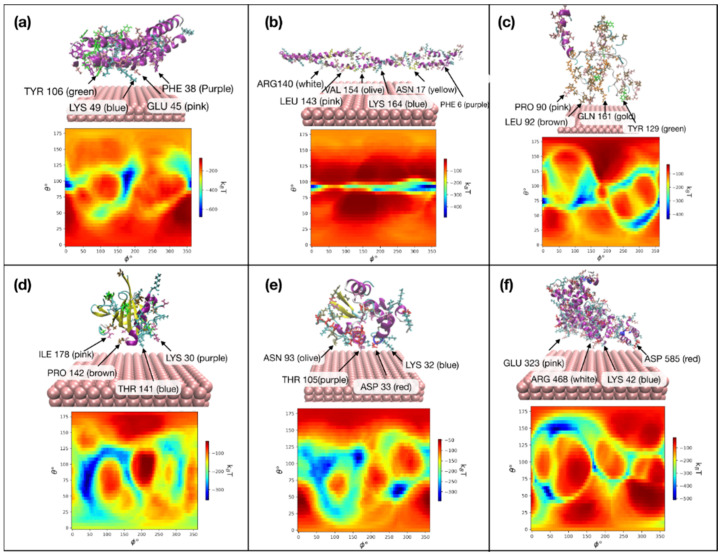
Preferred orientations (**top**) and adsorption energy heatmaps (**bottom**) of various milk proteins bound to the surface of aluminum (Al (110) with a surface size of 80 nm and a zeta potential of −5 mV at pH 7.0: (**a**) AS1C, (**b**) AS2C, (**c**) BC, (**d**) BLAC, (**e**) ALAC, and (**f**) SA. Blue areas (lower energies) represent more favorable orientations of proteins. Amino acids close to the Al surface are highlighted. Reproduced with permission from [83], Beilstein-Institut, 2024.

Zhong et al. simulated the adsorption of SA onto nanoparticles precoated with proteins such as myoglobin and k-casein, showing a stronger binding of SA to myoglobin than to k-casein whose binding domain was less exposed due to the specific covalent bonds between nanoparticle core and Asn and Gln of k-casein, suggesting that k-casein-coated nanoparticles had better antifouling properties because of their reduced protein corona formation [80]. Jomhori Baloch et al. compared the extents of the unfolding of insulin on copper nanoparticles with different sizes of 2, 6, and 10 nm, showing that insulin exhibited higher stability when interacting with the 10 nm copper nanoparticle surface compared to smaller nanoparticles. vdW forces increased from 2 nm to 6 nm but decreased for the 10 nm nanoparticle due to disulfide bonds and the reduced surface electron density [81]. Mekseriwattana et al. observed that the contact areas of both citrate-coated (C-SPION) and riboflavin-citrate-coated (Rf-SPION) superparamagnetic iron oxide nanoparticles were similar, despite the larger size of the riboflavin-citrate molecule. The lower free energy of solvation for C-SPIONs indicated a more hydrophilic surface compared to Rf-SPIONs [82]. Amini et al. investigated the binding of various milk proteins to pure aluminum surfaces, showing that BC and BLAC proteins favored aluminum surfaces (Al (100) and Al (110)), while AS1C protein preferred iron surfaces (Fe (100) and Fe (110)) (Figure 11) [83]. Yin et al. simulated SA and apolipoprotein E adsorbed onto PEGylated gold nanoparticles of varying surface curvatures, showing that as curvature increased, the adsorption surface area and strength of proteins decreased at smaller curvatures but increased at larger curvatures. Apolipoprotein E, with a regular shape, exhibited decreased adsorption strength with increasing curvature, while SA, with an irregular shape, showed increased adsorption strength due to the larger binding spaces provided by its structural junctions. This implied that nanoparticle curvature influenced protein adsorption in a non-monotonic manner, supporting experimental findings that showed a non-monotonic relationship between particle size and protein adsorption [84].

## 5. Interactions Among Lipids, Membranes, and Nanoparticles with Protein Corona

The presence of a protein corona on the nanoparticle surface modulates interactions between nanoparticles and lipid membranes, to an extent dependent on the electrostatics of lipid headgroups and proteins [85,86,87]. In particular, the protein corona can enhance or suppress the cellular uptake and internalization of nanoparticles [88,89], making it important to understand the interactions between membranes and nanoparticles with a protein corona. When nanoparticles interact with cell membranes or pulmonary surfactants, lipids can also adsorb to nanoparticle surfaces, forming a lipid monolayer or bilayer on the particle surface, referred to as the “lipid corona” [90,91,92]. The lipid corona has been relatively less studied compared to the protein corona, but recent research has intensively focused on it. Most simulations have been performed using CG models, which can capture the insertion of the nanoparticle–protein complex into the membrane and membrane curvature on a larger scale.

### 5.1. Mesoscopic and Coarse-Grained Simulations

**Figure 12:**
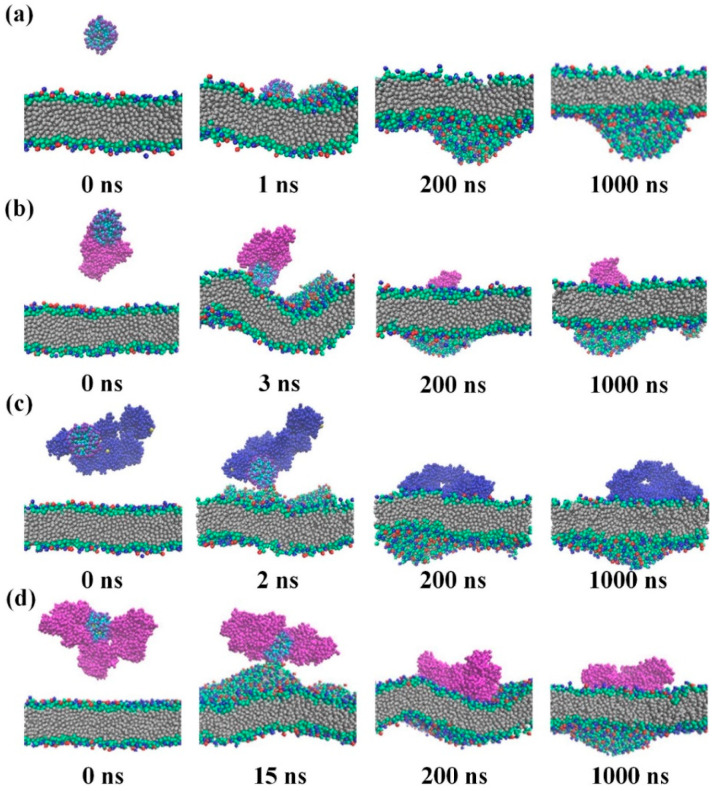
Interactions between membranes and various nanoparticles such as (**a**) a PEGylated nanoparticle, (**b**) a PEGylated nanoparticle with SA, (**c**) a PEGylated nanoparticle with complement C3b, and (**d**) a PEGylated nanoparticle with three SA proteins. Reproduced with permission from [96], Elsevier Ltd., 2023.

He et al. explored the adsorption mechanisms between patchy nanoparticles and SA, as well as the nanoparticle–SA interaction with lipid membranes, showing that SA preferentially interacted with the hydrophobic or charged regions of patchy NPs, with adsorption sites dependent on nanoparticle surface properties. The nanoparticle complexed with SA more strongly interacted with the lipid membrane than the nanoparticle alone did, implying that protein adsorption could enhance the nanoparticle–membrane binding [93]. Our group simulated the adsorption of lipids onto gold nanoparticles coated with phenylalanine (Phe), showing the formation of a lipid corona on the surface of gold nanoparticle via electrostatic interactions between charged lipid headgroups and terminal groups of phenylalanine. At low lipid concentrations, unsaturated lipids could form a complete lipid corona around a single gold–Phe nanoparticle, as observed in the experimental observation of lipid corona formation, while saturated lipids only partially coated all gold–Phe nanoparticles, causing them to aggregate [94]. Bai et al. found that the surface charge of nanoparticles significantly influenced their interactions with zwitterionic and anionic lipids, affecting how the lipid bilayer wrapped around the nanoparticle. In addition, differences in lipid composition between the corona and the bulk lipid structure were driven by the nanoparticle’s lipid affinity and the intrinsic properties of the lipids themselves [95]. Li et al. found that the pre-adsorption of SA onto nanoparticles weakened the interactions between nanoparticles and complemented protein C3b and the nanoparticle–membrane binding (Figure 12). This supported experimental observations that SA corona on the nanoparticle surface could inhibit further protein adsorption, thereby prolonging the circulating lifetime [96]. Angelescu et al. explored the formation of a chitosan corona around a polyvinyl chloride nanoparticle and the interaction of this complex with lipid membranes, showing that chitosan chains with high and intermediate protonation degrees spontaneously formed soft and hard coronas, which influenced the interaction of the polyvinyl chloride nanoparticle core with model membranes [97].

### 5.2. All-Atom Simulations

Our group calculated the free energies for the binding between lipid membranes and proteins adsorbed onto a nanoparticle, showing that the lateral dynamics of lipids was restricted by hydrogen bonds between proteins and zwitterionic membrane leaflets, while the electrostatic attraction between proteins and anionic membrane leaflets destabilized the helical structure of proteins and disordered membrane lipids. Also, protein corona formation suppressed the binding between particles and lipid membranes, in agreement with experiments, indicating the effect of the protein corona on membrane dynamics and protein–membrane binding strength [98].

## 6. Conclusions

Advances in computer power and simulation methodologies now allow for the accounting of atomic-level phenomena in protein corona formation using MD simulations. Simulations of mixtures involving multiple nanoparticles and proteins interacting with membranes have become feasible, utilizing both CG and all-atom models. These simulations provide valuable physical insights into the structure, dynamics, and composition of the protein corona, as well as its interactions with membranes, depending on factors such as protein type and concentration, ionic strength, and nanoparticle size, morphology, and surface properties. These interactions are governed by vdW, electrostatic, hydrogen-bonding, and hydrophobic interactions.

In addition to density functional theory and MD simulations, artificial intelligence (AI)-based high-throughput computational tools, such as machine learning, have recently been applied to predict the composition of the protein corona and its cellular recognition [99,100]. These multiscale computational approaches not only reproduce experimental observations and provide a deeper understanding of fundamental phenomena but also accurately predict protein corona formation and its effect on drug delivery efficiency, offering insights for manipulating it in the rational design of nanomedicines for drug delivery applications.

## Data Availability

Not applicable.
